# The current state of Japanese soybean breeding using DNA marker technology

**DOI:** 10.1270/jsbbs.25075

**Published:** 2026-02-28

**Authors:** Shin Kato, Naoya Yamaguchi, Akito Kaga

**Affiliations:** 1 Institute of Crop Science, National Agriculture and Food Research Organization (NARO), 2-1-2 Kannondai, Tsukuba, Ibaraki 305-8518, Japan; 2 Hokkaido Research Organization Central Agricultural Experiment Station, Higashi 6 sen Kita 15 Gou, Naganuma-cho, Yubari, Hokkaido 069-1395, Japan

**Keywords:** Japanese soybean, breeding program, MAS, identified genes, genomic selection, tolerance, seed quality

## Abstract

Following the recent global progress in the establishment of soybean genomic resources, DNA marker technologies have been actively implemented in soybean breeding programs in Japan, thereby enhancing the efficiency of varietal improvement and facilitating the successive release of cultivars developed through the application of DNA markers. In particular, DNA markers developed for the selection of useful traits, such as pod-shattering resistance and bacterial leaf pustule resistance present in foreign germplasms, have facilitated the precise introgression of only the desired alleles from genetically divergent foreign germplasms into Japanese backgrounds. By increasing selection efficiency and shortening breeding cycles, these advances have substantially contributed to the recent improvements in Japanese soybean breeding. The present review consolidates findings on genetic variations identified in Japanese cultivars and breeding materials, which have been investigated for the development of such DNA markers, and from knowledge expected to contribute to future soybean breeding in Japan.

## Introduction

Owing to its high protein and oil content, soybean [*Glycine max* (L.) Merr.] is the most important legume worldwide for food and industrial materials. It is also an important source for the production of traditional Japanese foods such as tofu, natto, miso, boiled beans, soy sauce, and edamame. In 2021, the demand for soybeans in Japan was approximately 3,540,000 tons, and that for related food use was approximately 1,030,000 tons per year (MAFF 2023). Therefore, almost all soybean produce, approximately 252,000 tons produced in Japan, is used for food, accounting for 24% of the soybean demand. Considering food security and the global protein crisis, the Ministry of Agriculture, Forestry, and Fisheries (MAFF) of Japan set an ambitious government target for domestic soybean production: 390,000 tons by 2030 in its Basic Plan for Food, Agriculture and Rural Areas formulated in 2025 (MAFF 2025). Assuming the current yield levels (161 kg/10a; average yield from 2019 to 2023), increasing soybean production by approximately 130,000 tons within the next five years would require an expansion of the current cultivation area (approximately 154,700 ha, MAFF 2023) by approximately 86,000 ha, which is extremely challenging. Therefore, enhancing cultivation management practices and improving soybean yield potential to increase production per unit area are essential. Furthermore, effectively incorporating seed quality traits and compositional factors identified in previous studies into domestic soybean breeding programs is crucial. Genetic control of seed appearance, quality, and composition leads to improvement in seed grade under the official agricultural inspection and adds value in terms of taste and functionality. These positive effects can lead to higher market prices and, ultimately, enhanced profitability for producers, thereby encouraging increased motivation for planting. This review summarizes the contributions of DNA markers and genomic information to addressing these challenges through breeding, and explores their potential future applications.

## Practical application of marker-assisted selection (MAS) in soybean breeding in Japan

**Fig. 1.**
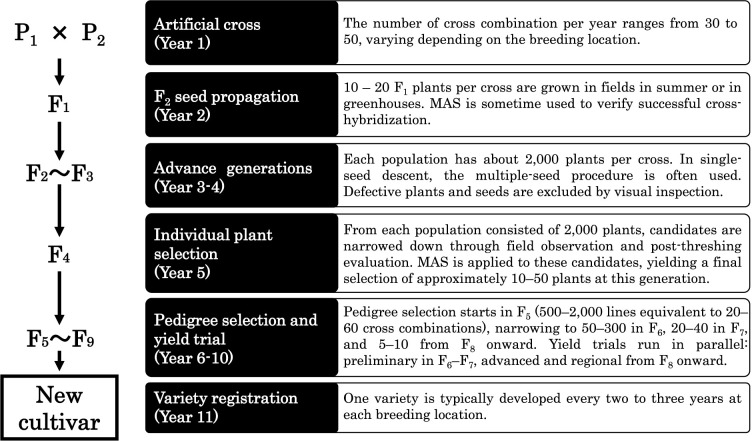
The conventional soybean breeding process and MAS in Japanese public institutions.

The most popular cross-breeding methods in Japan are mass breeding (Fig. 1). Artificial hybridization is carried out with the number of crosses ranging from approximately 30 to 50 per year. MAS is sometime applied to F_1_ seeds to verify successful cross-hybridization. The size of the F_2_–F_3_ populations is approximately 2,000 plants per cross. In single-seed descent, the multiple-seed procedure is often employed: one to two pods are harvested from each plant, and the pods are bulked together and threshed. Individual selection is generally conducted in the F_3_ or F_4_ generation, with field selection based on traits such as lodging resistance, plant architecture, maturity, and number of pods. After harvest, promising plants are further selected based on seed size, external seed quality, incidence of defective seeds, and protein content. Pedigree selections start from the F_4_ or F_5_ generation, and the total number of lines across all cross combinations in the F_5_ generation ranges from 500 to 2,000. MAS is applied at these stages: individual selection from the population, mainly the F_4_ generation, and first year of line cultivation, mainly the F_5_ generation. In early generations such as F_2_ and F_3_, typically 1,000 to 2,000 individuals are cultivated per cross combination. Because of the large population size, both sampling and genotyping are labor-intensive and inefficient; consequently, MAS is rarely implemented at this stage.

From the F_6_ generation onward, five plants are harvested individually from each selected line resulting in five lines that comprise the next generation’s line group. Yield trials are also conducted in parallel with line selection. The preliminary yield trials are conducted from the F_5_ to F_7_ generation, whereas advanced yield trials and multiple regional tests are initiated in the F_8_ generation and conducted in subsequent generations. Applications for plant variety registration and the production of breeder’s seed are most often carried out from the F_10_ generation onward. For plant variety registration, approximately one variety is commonly developed every two to three years at each breeding location.

Traditional MAS methods primarily rely on polyacrylamide or agarose gel electrophoresis. However, as described above, the need to process a large number of samples annually has led to the increasing adoption of more efficient technologies such as capillary electrophoresis systems and DNA sequencers. In particular, fragment analysis using DNA sequencing enables the simultaneous identification of multiple PCR products in a single reaction using primers labeled with four distinct fluorescent dyes, which significantly enhances throughput and efficiency (Kato *et al.* 2017, Sayama *et al.* 2011, Watanabe *et al.* 2018). However, the use of such advanced technologies involves substantial operational costs, particularly related to equipment maintenance and the employment of specialized technicians. In addition, given the limited manpower available to breeders, performing MAS in addition to core breeding activities is often impractical, both in terms of time and labor. In this context, the division of labor in MAS should become increasingly common, with specialized teams or units handling genotyping tasks separately from field breeding.

## Distinctive features of soybean breeding in Japan compared to international practices

**Fig. 2.**
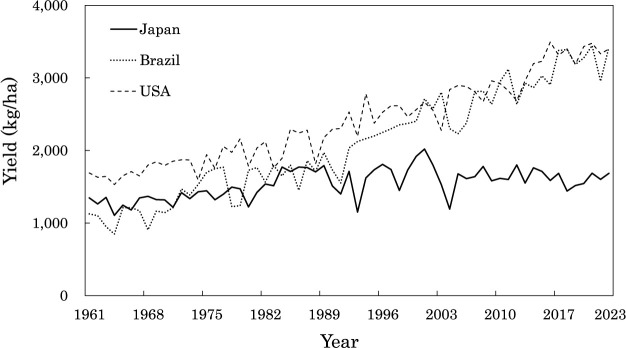
Trends in soybean yields in the United States, Brazil, and Japan (FAOSTAT).

Japanese soybeans have for a long time been subjected to selection for use as food and exhibit different characteristics from overseas soybeans. Seed characteristics for food processing, particularly for tofu, natto, and boiled beans, are important targets in soybean breeding in Japan, and seed quality and high protein content have been examined thoroughly (Escamilla *et al.* 2019, Taira 1990). In contrast, the focus for soybean breeding in other countries, such as the United States and Canada (hereafter, North America), and Brazil, has been improving its oil content and yield (Morrison *et al.* 2000, Tamagno *et al.* 2022, Umburanas *et al.* 2022). The yield gap between Japan and major soybean-producing countries such as the United States and Brazil began to widen around the 1990s, and in recent years has exceeded a twofold difference (Fig. 2). Boehm Jr. *et al.* (2019) has been pointed out that the increase in soybean yield in the United States has been largely driven by genetic improvement, and that this yield gain has come at the expense of seed protein content.

Although the scale of breeding differs among countries, the fundamental breeding processes are largely similar to those used in Japan. Nevertheless, the three major points of divergence are outlined below.

The first is the duration of cultivar development. As noted earlier, in Japan, it generally takes more than 10 years from the initial cross to cultivar release, whereas in North America, the process is typically completed in approximately 7 years (Vieira and Chen 2021). This acceleration is achieved by advancing the F_2_ and F_3_ generations in warm off-season nurseries located in the Southern Hemisphere, such as Chile and Puerto Rico.

The second point is the process of breeder seed production. In Japan, from approximately the F_5_ generation onward, repeated line selection is conducted over multiple years to increase genetic uniformity. Conversely, in North America, the process is relatively streamlined: promising plants are first selected from the population in yield trials. Seeds from each selected plant are then multiplied in bulk for each line while confirming that the resulting line is genetically uniform. The resulting line-level seeds are sown, and the genetic uniformity of each family is re-evaluated. If no uniformity issues are detected, all the families are harvested in bulk to produce breeder seeds. Given Japan’s stringent variety registration system, the bulk method for breeder seed production is not easily applicable. However, the North American system allows for a smoother transition from breeding to seed production, and, ultimately, to seed delivery for growers.

The third point concerns the yield evaluation methods. In North America, mechanical harvesting using plot combines has long been a standard practice, whereas in Japan, yield testing has traditionally been dependent on manual harvesting. Therefore, the methodological difference between yield assessment in breeding facilities and actual farmers’ field harvesting practices may have contributed to the yield gap observed between the Japanese and North American cultivars. In Japan, mechanical harvest-based yield evaluation has historically lagged, largely because of the predominance of paddy-field conversion farmlands and limited field size. However, with the urgent need to develop high-yield cultivars, mechanized yield testing has recently been introduced. Indeed, cultivars such as ‘Sorahibiki’ (Shimamura *et al.* 2025), ‘Soramizuki’ (Kato *et al.* 2023), ‘Soratakaku’ (Sayama *et al.* 2025) and ‘Soraminori’ (Oki *et al.* 2025) have been released, demonstrating the effectiveness of this approach. MAS remains challenging for key agronomic quantitative traits such as yield, protein content, and lodging resistance. Consequently, breeders must continue to rely on field-based evaluations, visually assess individual soybean plants for these important traits, and make the appropriate selection decisions. Thus, it is crucial to distinguish between traits that are suitable for MAS and those that are more effectively addressed through phenotypic selection, such as yield evaluation using mechanical harvesting.

## Development of reference genomes

The first soybean reference sequence for the US soybean cultivar ‘Williams 82’ was released in 2010. In the latest version, approximately 53,000 genes were predicted across roughly 978 million base pairs (Schmutz *et al.* 2010). Since then, high-quality reference genomes have been successively published from various countries, including the US cultivar ‘Lee’, a Chinese wild soybean (Valliyodan *et al.* 2019), the Chinese cultivar ‘Zhonghuang 13’ and wild soybeans (Shen *et al.* 2019), 23 cultivated and 3 wild soybeans (Liu *et al.* 2020), the Korean cultivar ‘Hwangkeum’ (Kim *et al.* 2021), the Chinese cultivar ‘Jidou 17’ (Yi *et al.* 2022), the Chinese vegetable soybean cultivar ‘Zhenong 6’ (Liu *et al.* 2022), telomere-to-telomere gap-less genome assemblies of the Chinese cultivar ‘Yundou1’, and wild soybean ‘Yesheng71’ (Jia *et al.* 2024). Recently, Yano *et al.* (2025) constructed a nanopore-based genome reference for seven Japanese and four overseas soybean cultivars. Notably, a high-quality reference genome with 58,646 genes was constructed for the cultivar ‘Enrei’, which serves as the reference standard for domestically produced soybeans. Although the availability of precise genomic information has advanced significantly, the number of genes with clearly defined functions remains limited. Functional annotations have been assigned to numerous soybean genes; however, most of them are based on predictions derived from homologous genes in other organisms and model plant species. Agronomically and industrially important traits such as yield and seed quality are regulated by complex interactions among numerous genes, further limiting the number of genes whose functions have been definitively characterized.

## Utilization of DNA markers in soybean breeding

Agriculturally important genes are difficult to directly identify; even so, their approximate chromosomal locations can be inferred by mapping techniques using DNA markers and quantitative trait locus (QTL) analysis. These approaches allow for MAS of desirable traits without requiring full elucidation of the underlying genes. SoyBase (https://legacy.soybase.org/), developed by USDA, is the world’s largest integrated database for soybean breeding information. It provides searchable chromosomal location data for 7,246 loci and QTLs associated with 909 traits, thereby considerably facilitating the design of precise DNA markers. High-quality DNA markers capable of reliably detecting the presence or absence of beneficial traits have significantly contributed to selection efficiency and shortening of breeding cycles. In recent years, DNA markers have played a crucial role in the targeted improvement of soybean cultivars in Japan; however, in many cases, their use in cultivar development has not been thoroughly documented, and comprehensive reports on the application of MAS in Japanese soybean breeding remain scarce. The following sections present the identified genes, as summarized in Supplemental Table 1, and underlying mechanisms for each trait as well as the DNA markers and cultivars developed through their application in MAS.

## Flowering and maturity

Soybeans are cultivated across the Japanese archipelago, which spans a wide range of latitudes. Therefore, selecting cultivars that have adapted to the specific photoperiod and temperature conditions of each region is essential. In northern Japan, where snowfall occurs, the ability of a cultivar to mature before the onset of snow is a particularly critical trait. Moreover, the duration of the vegetative growth phase prior to floral induction significantly influences regional adaptability as well as plant architecture, biomass accumulation, and ultimately yield. Since the 2000s, the availability of genomic information has enabled elucidation of the molecular mechanisms underlying approximately 18 major genes related to flowering and maturity.

Among these, the *E1* gene is known to have the most substantial effect on flowering time. It encodes a transcription factor with a B3 domain and is considered to be a legume-specific gene (Xia *et al.* 2012), absent in model plants such as rice and *Arabidopsis*. The expression of *E1* is regulated by the *ELF* gene (Lu *et al.* 2017, classical *J* locus) and the *LHY* gene (Dong *et al.* 2021), which in turn control the downstream florigen genes *GmFT2a* (Kong *et al.* 2010) and *GmFT5a* (Takeshima *et al.* 2016). Xia *et al.* (2012) reported multiple alleles at the *E1* locus, including *e1-nl* (a complete deletion resulting in the loss of photoperiod sensitivity), *e1-fs* (a frameshift mutation), and *e1-as* (a missense mutation with reduced function). Cultivars lacking all four dominant alleles—*E1*, *E2*, *E3*, and *E4*—have been identified in Hokkaido. Additionally, homologs of *E1*, namely *E1La* (Xu *et al.* 2015) and *E1Lb* (Zhu *et al.* 2019), participate in the regulation of florigen gene expression.

The *E2* gene corresponds to the GIGANTEA gene, a signal transduction factor in the circadian clock (Watanabe *et al.* 2011). Loss-of-function mutations in *E2* result in early flowering, and such alleles are commonly found in cultivars from Honshu. The *E3* and *E4* genes encode PHYA3 (Watanabe *et al.* 2009) and PHYA2 (Liu *et al.* 2008), respectively. Loss-of-function mutations lead to early flowering. Cultivars adapted to high-latitude regions, such as Tohoku and Hokkaido, often carry mutations in *E4*, whereas loss-of-function alleles of *E3* are frequently observed in Honshu cultivars; in addition, DNA markers for *E2*, *E3* and *E4* have been developed (Tsubokura *et al.* 2014).

*Tof11* and *Tof12* encode pseudo-response regulators (PRRs) that mediate signal transduction in response to environmental light cues (Lu *et al.* 2020). Most Japanese soybean cultivars possess a non-functional *Tof12* allele and a functional *Tof11* allele. In contrast, some foreign cultivars carry non-functional *Tof11* alleles, which affect flowering time and shorten the reproductive growth phase.

Florigen genes *GmFT2a* and *GmFT5a* promote floral induction under short-day conditions. The maturity gene *E9* is *FT2a*, and its recessive allele delays flowering because of its lower transcript abundance, which is caused by allele-specific transcriptional repression owing to the insertion of *SORE-1* (Zhao *et al.* 2016). Regardless of the day length, the *e9* allele delayed flowering. Hokkaido cultivars carry this mutation.

The *J* gene (*ELF* gene) is an allele found in cultivars adapted to low-latitude regions that delay flowering under short-day conditions (Lu *et al.* 2017). Under these conditions, the *J* gene suppresses *E1* expression, allowing for the expression of florigen genes and floral induction. When the *J* gene loses its function, *E1* expression is no longer suppressed under short-day conditions, resulting in delayed flowering. The *LUX* gene, which forms a complex with the *J* gene, has been identified as a component of the Evening Complex in soybean (Bu *et al.* 2021).

As noted above, flowering and maturity are controlled by multiple genes; thus, materials exhibiting similar phenotypes may nonetheless possess different genetic compositions. However, the genes described above allow a substantial degree of predictive inference, and this information will facilitate detailed genotyping of breeding materials in the future. Consequently, if crossing combinations that are unlikely to exhibit substantial segregation for maturity in their progeny can be pre-selected, efficient yield evaluations can be conducted while minimizing the confounding effects of maturity variation.

## Plant architecture

Soybeans exhibit three distinct growth habits: determinate types, which cease stem elongation upon floral induction; indeterminate types, which continue stem elongation and leaf formation after flowering; and semi-determinate types, which show intermediate stem termination between determinate and indeterminate types (Bernard 1972). Determinate growth is common in Japanese cultivars, whereas indeterminate and semi-determinate growth are more prevalent in overseas varieties. The genetic background and environment determine whether indeterminate phenotypes have a higher yield than determinate phenotypes (Cober and Morrison 2010, Kato *et al.* 2015). Most soybean cultivars developed in high-latitude countries have an indeterminate growth habit; however, this is not found in any of the current commercial Japanese cultivars. ‘OAC Dorado’, a Canadian cultivar with an indeterminate growth habit, produced significantly greater yield than Hokkaido’s leading cultivar ‘Yukihomare’ in fields in Hokkaido, indicating that it may be possible to breed a high-yielding cultivar with an indeterminate growth habit (Yamaguchi *et al.* 2019b).

Growth habit is regulated by the interaction between the *Dt1* gene, which encodes the TFL1 protein that suppresses floral meristem formation (Tian *et al.* 2010), and the *Dt2* gene, which encodes a MADS-box transcription factor that binds to the promoter region of *Dt1* and acts as a repressor (Ping *et al.* 2014). Combinations of these alleles determine the growth habit: *dt1dt2* and *dt1Dt2* result in determinate growth, whereas *Dt1Dt2* and *Dt1dt2* lead to semi-determinate and indeterminate growth habits, respectively. The MAS for the *Dt1* allele is conducted in breeding programs mainly in Hokkaido.

Soybeans develop inflorescence meristems on each node, including the shoot apex. Raceme phenotype is an important trait associated with the number of floral buds and pods. *qTRL18-1*, a QTL associated with terminal raceme length, was detected in the proximal region of the *Dt2* locus (Yamaguchi *et al.* 2014a). Another QTL, *qTRL11-1*, was detected only in the *qTRL18-1* background, indicating an interaction between *qTRL18-1* and *qTRL11-1*. ‘Tokei 1122’, which has a long terminal raceme with *qTRL18-1* and *qTRL11-1*, was bred to improve the yield of low-branching cultivars under dense planting conditions. In the yield tests under dense planting conditions (33 plants m^–2^), the seed yield of ‘Tokei 1122’ was 34.8 kg/a, whereas ‘Toyoharuka’, a low-branching cultivar bred in Hokkaido, yielded 29.6 kg/a, representing an approximately 18% increase (Kitabatake *et al.* 2020).

## Lodging resistance

Lodging resistance is an important trait in soybean breeding because lodging significantly reduces cultivation management operations, mechanical harvest efficiency, light-use efficiency, soybean yield, and quality when plants fall over or lean excessively in the field due to typhoons and storms with heavy rain and wind (Saitoh *et al.* 2012). In Japan, poor drainage in converted paddy fields often lead to lodging after frequent rainfall and strong winds, including typhoons. Breeding for lodging resistance helps ensure crop growth stability under adverse weather conditions, which is increasingly important under climate variability.

Most of the investigated QTLs for lodging resistance in Japanese cultivars is located in the proximal region of the maturity or growth habit locus, *Dt1*. *qLS19-1* was identified using a population derived from a cross between ‘Toyoharuka’ (a lodging-tolerant cultivar) and ‘Toyomusume’ bred in Hokkaido (Yamaguchi *et al.* 2014b). The Toyoharuka allele at *qLS19-1* increases the number of primary lateral roots and causes high pushing resistance, resulting in a lower ratio of the pushing resistance moment (Kitabatake *et al.* 2019). Eight QTLs associated with seed yield were identified using a population derived from a cross between ‘Toyoharuka’ and ‘Toyomusume’ (Yamaguchi *et al.* 2021a). Six breeding lines pyramiding favorable alleles at the seven QTLs associated with seed yield and *qLS19-1* were developed from the same population, and their seed yields tended to exceed those of their parental cultivars (Yamaguchi *et al.* 2021b). ‘Toyomadoka’ is a lodging tolerant cultivar bred in Hokkaido, and has ‘Toyoharuka’ in its pedigree (Kobayashi *et al.* 2020). The marker analysis revealed that ‘Toyomadoka’ has the ‘Toyoharuka’ allele at *qLS19-1*, indicating that MAS for the ‘Toyoharuka’ allele at *qLS19-1* will be effective for improving lodging tolerance. Backcross breeding introducing the ‘Toyoharuka’ allele at *qLS19-1* to easily lodging cultivars is in progress, and the effects of *qLS19-1* will be validated in various genetic backgrounds in future.

A major QTL, designated *qSI13-1* on chromosome 13, was founded from a Japanese germplasm ‘Y2’ exhibiting significantly shorter internode lengths compared to modern Japanese cultivars (Oki *et al.* 2018). This QTL confers shorter plant stature and internode length without affecting the flowering time, node number, or seed yield. Advanced breeding lines developed through backcrossing using major cultivars such as ‘Fukuyutaka’ as recurrent parents have been developed, and their use in future breeding programs, as well as high-yield cultivation practices, is being considered. A robust lodging tolerance QTL, *qLT13-1*, was identified on chromosome 13, explaining 20% of phenotypic variance in lodging angle across different environments (Hishinuma *et al.* 2025). US cultivars-derived alleles at this locus consistently improve lodging tolerance under multiple conditions. Recently, an increased copy number of two gibberellin 2-oxidase 8 genes (*GmGA2ox8A* and *GmGA2ox8B*) in a close genomic region on chromosome 13 was identified as the gene responsible for reduced trailing growth and shoot length (Wang *et al.* 2021a). An increased copy number reduces gibberellin activity, resulting in shorter plant height and contributing to lodging resistance. Another gene, *PH13*, which regulates main stem length, was isolated from different genomic regions on chromosome 13 (Qin *et al.* 2023). This gene encodes a suppressor of the phyA-105 (SPA) protein, which suppresses photomorphogenesis as part of a complex with COP1, and truncated PH13 with reduced interaction with COP1, resulting in the accumulation of STF and reduced plant height. These genes found on chromosome 13 are likely to be associated with the lodging resistance QTL mentioned above, so it is necessary to verify the selection effect on lodging using DNA markers designed around these genes.

## Pod-shattering resistance

Pod-shattering resistance is a critical agronomic trait for enhancing soybean yield because it prevents seed loss resulting from natural pod dehiscence, particularly under conditions of delayed harvest (Funatsuki *et al.* 2008, Yamada *et al.* 2009). This trait is especially beneficial for mechanical harvesting as it minimizes seed loss during operations and contributes to yield stability (Ndeke *et al.* 2024, Yamada *et al.* 2017). Of these, *pdh1* is the most widely utilized in major soybean-producing countries such as the United States and Canada (Funatsuki *et al.* 2014). The *PDH1* gene encodes a dirigent-like protein involved in the regulation of pod wall torsional stress, which leads to pod opening (Funatsuki *et al.* 2014). Although this trait provides a clear advantage by reducing seed loss at harvest, it has been underutilized in Japan, where many cultivars still lack effective pod-shattering resistance, in contrast to the high prevalence of this trait in US cultivars. Therefore, using DNA markers for *pdh1*, new pod-shattering resistant varieties were developed through backcrossing with elite Japanese cultivars, ‘Enrei’, ‘Kotoyutaka’, ‘Sachiyutaka’, and ‘Fukuyutaka’ as recurrent parents. As a result, the pod-shattering resistant lines ‘Enrei no Sora’ (Yamada *et al.* 2013), ‘Kotoyutaka A1 gou’ (Yamada *et al.* 2013), ‘Sachiyutaka A1 gou’ (Hajika *et al.* 2016), and ‘Fukuyutaka A1 gou’ (Hajika *et al.* 2019) were developed. Although ‘Enrei no Sora’ showed a slightly later maturity compared to ‘Enrei’, no significant differences in agronomic traits or processing qualities were observed in the other varieties. In a yield comparison trial using combine harvesting between ‘Fukuyutaka’ and its pod-shattering resistant line ‘Fukuyutaka A1 gou’, the yield of ‘Fukuyutaka’ was 127.4 kg/10a, whereas ‘Fukuyutaka A1 gou’ yielded 183.8 kg/10a, representing an approximately 44% increase (Hajika *et al.* 2019). This yield advantage is attributed to reduced seed loss due to shattering, as the loss of ‘Fukuyutaka’ (based on small-plot sampling) was 91.3 kg/10a, compared to only 23.2 kg/10a for ‘Fukuyutaka A1 gou’, clearly demonstrating the effect of *pdh1* in reducing pod shattering. As of 2024, the cultivation areas of ‘Enrei no Sora’, ‘Kotoyutaka A1’, ‘Sachiyutaka A1’, and ‘Fukuyutaka A1’ were 3,408 ha, 2,326 ha, 1,514 ha, and 4,381 ha, respectively, and further expansion of their cultivation is expected in the future.

Phenotypic selection for pod-shattering resistance has been conducted since 1975 in Hokkaido, and some cultivars bred in Hokkaido have *pdh1*, including ‘Kariyutaka’ (Tanaka *et al.* 1993), ‘Hayahikari’ (Yumoto *et al.* 2000), ‘Yukihomare’ (Tanaka *et al.* 2003), ‘Yukihomare R’ (Suzuki *et al.* 2017), ‘Toyomizuki’ (Yamaguchi *et al.* 2023), and ‘Toyomadoka’ (Kobayashi *et al.* 2020). Particularly, MAS for *pdh1* was conducted in the F_4_ generation during the breeding process of ‘Toyomadoka’.

Two other genes have been identified in relation to pod-shattering resistance in soybeans: *SHAT1-5*, which encodes an NAC transcription factor (Dong *et al.* 2014), and *Sh1*, which encodes a C2H2-like zinc finger transcription factor (Li *et al.* 2024). The functional allele of *Sh1* promotes pod shattering by repressing the expression of *SHAT1-5*, which leads to reduced secondary wall thickness in the fiber cap cells of the abscission layer of pods.

However, 19.8% yield loss due to pod shattering has been reported, even for varieties with *pdh1* and *SHAT1-5* resistant alleles (Takahashi *et al.* 2023, Yamada *et al.* 2017). Generally, delays in harvesting and environmental conditions, such as high temperature and low humidity, increase the risk of pod shattering (Ndeke *et al.* 2024). The soybean cultivation area per farming corporation is expanding, and with ongoing climate change, the development of new genetic controls to improve pod-shattering resistance is highly anticipated.

## Seed size

Increasing soybean seed size is a critical trait in breeding because of cultural preferences and market demand in Japan. Larger seeds are particularly valued by food manufacturers for traditional food products such as tofu and boiled soybeans. Because seed size can be readily evaluated visually in practical breeding programs in Japan, MAS has rarely been employed, even though seed size is a key trait in domestic soybean breeding. Soybean seed size is a typical quantitative trait controlled by multiple loci with small effects, and genes controlling maturity and oil and protein content also influence seed size (Duan *et al.* 2023). However, only a few causal genes of natural variations related to seed size have been identified. Zhang *et al.* (2024) summarized the molecular regulatory network controlling seed size in soybeans, and more than 30 genes have been reported to regulate seed size. *GA3ox1* encodes gibberellin 3β-hydroxylase, and reductions in individual seed size are observed in lines harboring mutations in this gene, despite the concomitant increase in seed yield driven by higher seed number (Hu *et al.* 2022). *GmSW17* (*Seed Width 17*) on chromosome 17 controls seed width and weight, and encodes ubiquitin-specific protease affecting cell expansion and cell division (Liang *et al.* 2024). *GmKIX8-1* encodes a protein containing a KIX domain that suppresses cell proliferation, and loss-of-function mutant plants show an increased seed size (Nguyen *et al.* 2021). Differences in the copy number of the CT-core microsatellite motif in the 5ʹ-untranslated region of *WRKY15a* are associated with gene expressions of this gene and seed sizes (Gu *et al.* 2017). To facilitate seed size control and enhance breeding efficiency in the future and enable the accurate prediction of seed size distributions in segregating progenies based on parental allele combinations, a comprehensive catalog of genes controlling seed size across a wide range of breeding materials, including varieties, breeding lines, and genetic resources will have to be compiled.

## Protein

Soybean seeds accumulate approximately 40% protein, 20% lipids, and 35% carbohydrates (Cheftel *et al.* 1985) and are utilized as a raw material in the food and feed industries. Approximately 70% of soybean seed protein comprises two major storage proteins: glycinin (11S globulin) and β-conglycinin (7S globulin) (Hill and Breidenbach 1974, Thanh and Shibasaki 1978). A trade-off exists between glycinin and β-conglycinin contents, influencing food processing properties (Yang *et al.* 2016). ‘Yumeminori’ (Takahashi *et al.* 2004) and ‘Nagomimaru’ (Hajika 2009), which lack 7S α and αʹ subunits and show increased glycinin content, and ‘Nanahomare’, which lacks most glycinin subunits and accumulates β-conglycinin (Yagasaki *et al.* 2013) have been developed. Experimental lines like ‘QF2’ lack both β-conglycinin and glycinin but compensate with free amino acids (Takahashi *et al.* 2003). Selection of these soybean cultivars lacking seed storage proteins has relied on sodium dodecyl sulfate polyacrylamide gel electrophoresis (SDS-PAGE) analysis. However, selections on SDS-PAGE are labor-intensive and sometimes fail to separate glycinin subunits clearly leading to phenotyping errors due to visual judgment. Therefore, the development and application of DNA markers for these storage proteins offer a more reliable and efficient approach for stable evaluation and large-scale selection of breeding lines.

Glycinin is composed of five subunits: A_1a_B_2_, A_1b_B_1b_, A_2_B_1a_ (Group I), A_3_B_4_, and A_5_A_4_B_3_ (Group II) (Nielsen 1985), and is encoded by five genes: *Gy1* (A_1a_B_2_), *Gy2* (A_2_B_1a_), *Gy3* (A_1b_B_1b_), *Gy4* (A_5_A_4_B_3_), and *Gy5* (A_3_B_4_) (Nielsen *et al.* 1989). In addition, the presence of two pseudogenes, *Gy6* and *Gy8*, and one gene with weak expression activity, *Gy7*, has been confirmed (Li and Zhang 2011). On the other hand, 7S globulin consists of three subunits: α, αʹ, and β, and were reported to be encoded by a family of 15 genes, *CG-1* to *CG-15*. *CG-1* (αʹ-subunit), *CG-2* (α-subunit), *CG-3* (α-subunit), and *CG-4* (β-subunit) have been identified, whereas whether genes *CG-5* to *CG-15*, which have been reported as genes, are functional is unclear (Harada *et al.* 1989, Singh *et al.* 2015). In addition, the mutation that lacks all 7S globulin subunits found in wild soybean ‘QT2’ is controlled by the dominant *Scg-1* mutation (Hajika *et al.* 1998). *Scg-1* is located on chromosome 20, and post-transcriptional silencing occurs owing to the inverted repeat sequence of the α subunit gene, suppressing the expressions of the β-conglycinin genes (Tsubokura *et al.* 2012). DNA markers for the selection of these genotypes have been developed (Sayama *et al.* 2015), and their implementation in practical breeding programs is in progress.

Protein and oil contents are negatively correlated due to competition for carbon skeletons, and increasing seed number reduces assimilate concentration per seed, creating a negative correlation between protein content and yield—major obstacles for breeding high-yield, high-protein soybeans. Guo *et al.* (2022) summarized QTL information for soybean seed protein content in SoyBase. In total, 249 QTLs were detected on all 20 chromosomes; however, stable and consistent QTL with considerable effects is limited. According to Patil *et al.* (2017), the Soybean Genetics Committee has officially confirmed *cqPro-15* on chromosome 15 and *cqPro-20* on chromosome 20 as stable QTLs in many populations. Causal genes for natural variations in protein content have been identified on chromosomes 15 and 20. *GmSWEET10a*, located on chromosome 15, encodes a sucrose transporter (Wang *et al.* 2020). When a mutation occurs in the promoter region, the transcription level increases, leading to increased transport of sucrose and hexose, which are carbon sources for lipids, to the seeds, thereby increasing lipid content and seed size and decreasing protein content. *POWR1*, located on chromosome 20, encodes a protein that regulates lipid metabolism and nutrient transport (Goettel *et al.* 2022). Varieties with a transposon inserted into the CCT domain show altered downstream gene expression, resulting in increased lipid content and seed size and in decreased protein content. *POWR1* may have been utilized in the development of high-protein soybean cultivars in Japan. ‘Saikai 20’ (PI 423949) is known as a high-protein breeding line with a high-protein allele of *POWR1* (Fliege *et al.* 2022), and has been used as a valuable parent in crosses, leading to the development of other promising high-protein lines and varieties such as ‘Tomutan’. These varieties were developed through selection based on the protein content measured using near-infrared spectroscopy. Although their sequences of *POWR1* have not been compared yet, pedigree information suggests that they may have inherited the high-protein allele from ‘Saikai 20’. As mentioned above, the development of high-protein cultivars has thus far been dependent on phenotypic evaluation using near-infrared spectroscopy; however, these findings are expected to contribute to MAS of high-protein varieties in the future.

## Fatty acids

Soybean oil contains approximately 13% α-linolenic acid (18:3), 55% linoleic acid (18:2), 18% oleic acid (18:1), 4% stearic acid (18:0), and 10% palmitic acid (16:0) (Clemente and Cahoon 2009). To improve the oxidative stability of soybean oil and its quality during high-temperature cooking, mutational breeding has been conducted to increase the proportions of oleic acid (18:1), a monounsaturated fatty acid. Oleic acid is converted to linoleic acid (18:2) by delta-12 fatty acid desaturase 2 (FAD2), which is encoded by two genes: *GmFAD2-1a* and *GmFAD2-1b* (Anai *et al.* 2008). In double mutants combining *GmFAD2-1a* and *GmFAD2-1b*, the oleic acid content exceeds 80% of total fatty acids, whereas polyunsaturated fatty acids are reduced to below 10% (Hoshino *et al.* 2010).

The biosynthesis of α-linolenic acid (18:3) is controlled by three genes encoding omega-3 fatty acid desaturases (*GmFAD3-1a*, *GmFAD3-1b*, and *GmFAD3-2a*) (Anai *et al.* 2005). In triple mutant lines combining loss-of-function alleles of these genes, the α-linolenic acid content in seeds is reduced to the 1% level of total fatty acids (Hoshino *et al.* 2014).

The double mutant line of *GmFAD2-1a* and *GmFAD2-1b* was developed as the soybean cultivar ‘Sadai HO1 gou’ in 2018 (Anai 2020). This cultivar exhibits five times higher oxidative stability compared with ‘Fukuyutaka’, and its extremely low linoleic acid content results in almost no formation of n-hexanal with undesirable green or rancid odor via lipoxygenase activity.

## Saponin

Soybean saponins are a group of glycosides composed of oleanane-type triterpenoid aglycones with various sugar moieties and more than 50 kinds of saponin molecules have been identified (Tsukamoto 2012). Group A saponins are responsible for undesirable bitter or astringent aftertastes (Okubo *et al.* 1992). In contrast, DDMP saponins and their derivatives (*e.g.*, group B and E saponins) derived from soyasapogenol B exhibit various health-promoting properties such as the prevention of dietary hypercholesterolemia and suppression of colon cancer cell proliferation (Yano *et al.* 2017). Therefore, genetical controls of saponin composition could improve both the taste and functionality of soybean-derived food products, ultimately enhancing the value of soybean cultivars.

To date, five genes: *Sg-1* (Sayama *et al.* 2012), *Sg-3* (Yano *et al.* 2018), *Sg-4* (Takagi *et al.* 2018), *Sg-5* (Yano *et al.* 2017), *and Sg-9* (Sundaramoorthy *et al.* 2019), have been identified, and the use of the mutant allele of *Sg-1* has been the greatest contributor to soybean breeding in Japan. The *Sg-1* gene encodes a UDP-sugar-dependent glycosyltransferase that mediates glycosylation of the terminal sugar at the C-22 position of group A saponins (Sayama *et al.* 2012). In the codominant alleles *Sg-1^a^* and *Sg-1^b^*, the glycosyltransferases UGT73F4 and UGT73F2 are functionally expressed, leading to the production of group A saponins with acetylated xylose (Aa-type) and acetylated glucose (Ab-type), respectively, as terminal sugars. The key functional distinction between *Sg-1^a^* and *Sg-1^b^* is the amino acid at position 138, which is Ser in *Sg-1^a^* and Gly in *Sg-1^b^*; this difference is critical for defining their sugar donor specificities. Conversely, *sg-1^0–a^* and *sg-1^0–b^* are loss-of-function alleles of *Sg-1^a^* and *Sg-1^b^*, respectively, resulting in the production of A0-type saponins that lack acetylated terminal sugars. Because acetylated sugars are considered responsible for imparting undesirable bitterness and astringency, processed soybean products such as soymilk and tofu made from A0-type varieties have been evaluated as having superior flavor quality. Based on this characteristic, varieties with A0-type group A saponin, such as ‘Kinusayaka’ (Kato *et al.* 2007) and ‘Sumisayaka’ were developed and registered in 2008 and 2024, respectively. During the development of these cultivars, MAS using SSR markers located near *Sg-1* was partially employed in conjunction with phenotypic evaluation based on thin-layer chromatography. These cultivars are mainly used for soymilk production and were cultivated on approximately 94 ha in the Tohoku region and 155 ha in the western region of Japan by 2023.

## Boiled seed hardness

Boiled seed hardness is an important factor in the processing of soybean food products, not only for cooked beans but also for miso and natto, which necessitate bean softening by steaming or boiling prior to fermentation (Motoki *et al.* 2013, Yoshioka *et al.* 2009). ‘Hyoukei-kuro 3’ is a Japanese soybean variety known for its soft boiled seed texture after steaming. Hirata *et al.* (2014) have identified *qHbs3-1* and *qHbs6-1* associated with boiled seed hardness on chromosomes 3 and 6 using RILs developed from a cross between ‘Natto-shoryu’ (hard) and ‘Hyoukei-kuro 3’ (soft). Subsequent progress in positional cloning and related studies led to the identification of pectin methylesterase (PME) as the causal gene of *qHbs3-1* (Toda *et al.* 2015). PME de-esterifies pectin in cell wall by converting methoxyl to carboxyl groups, releasing methanol and protons (H^+^). Carboxyl groups cross-link with Ca^2+^, increasing cell wall rigidity and boiled seed hardness. The ‘Hyoukei-kuro 3’ allele introduces a premature stop codon, producing a truncated PME lacking activity, likely preventing hardness during boiling. Additionally, F3ʹH, the gene underlying the T locus, has been identified as the causal gene for *qHbs6-1* (Toda *et al.* 2022). Ca^2+^ and Mg^2+^ contents of varieties with F3ʹH active allele (*e.g.*, ‘Hyoukei-kuro 3’) were higher in pods and lower in seeds than those with inactive allele (*e.g.*, ‘Natto-shoryu’), suggesting that F3ʹH inhibits the transport of Ca^2+^ and Mg^2+^ from pods to seeds as well as increase in heat-labile pectin caused by fewer cross-linked divalent ions, and softens boiled beans.

Effective amplification refractory mutation system (ARMS) markers for PME were developed by Toda *et al.* (2020). Although there are no reported cases where it has been directly introduced into leading varieties through backcrossing, it is utilized in population selection and early generation line selection, particularly for breeding varieties suitable for natto and boiled beans. In contrast, F3ʹH has been reported to exhibit pleiotropic effects, including suppression of Ca^2+^ and Mg^2+^ translocation, as well as influence on pubescence color, seed coat color, and chilling tolerance (Cober *et al.* 1998, Yamaguchi *et al.* 2021c). Among these, seed coat color is particularly problematic; when combined with the *I* locus in the *IITT* genotype, the yellow seed coat becomes dark, resulting in an undesirable seed appearance. Therefore, in contrast with PME, F3ʹH is not actively utilized in breeding.

## Seed flavor

Lipoxygenases (LOXs) in soybean seeds are enzymes that catalyze the peroxidation of polyunsaturated fatty acids and the generation of volatile compounds, such as n-hexanal, which cause undesirable flavors in soybean products. The absence of LOX1, LOX2, or LOX3 in seeds is controlled by the single recessive alleles, *lx1* (Hildebrand and Hymowitz 1981), *lx2* (Davies and Nielsen 1986), and *lx3* (Kitamura *et al.* 1983), respectively. Hajika *et al.* (1991) developed a mutant lacking all lipoxygenases by gamma-ray irradiation and the first lipoxygenase-less variety, named ‘Ichihime’, was developed using the progeny of this mutant (Hajika *et al.* 2002, 2009). Several lipoxygenase-less Japanese cultivars including ‘L-star’ and ‘Suzusayaka’ have been developed.

Previously described, lipoxygenase-deficient varieties have primarily been selected through phenotyping based on SDS-PAGE analysis. In addition, in segregating populations, the selection of deficient individuals has relied largely on a simple and rapid screening method based on the bleaching activities of these isozymes developed by Suda *et al.* (1995) owing to financial and labor constraints. Consequently, the development and application of DNA markers remain limited. However, because all lipoxygenase-deficient traits exhibit recessive inheritance, distinguishing between wild-type and heterozygous genotypes based solely on phenotypes is impossible. Therefore, MAS is particularly effective during backcrossing, when elite cultivars are used as recurrent parents. The genes responsible for LOX1, LOX2, and LOX3 have been isolated and are located on chromosomes 13, 13, and 15. Notably, *Lx1* and *Lx2* are positioned in close proximity and approximately 3 kb apart. In Japanese breeding programs, the mutant alleles *lx1*, *lx2*, and *lx3* are derived from ‘PI 408521’ (premature stop codon caused by a 74bp deletion), ‘PI 86023’ (missense mutation substituting Gln for His-532), and ‘PI 205085’ (premature stop codon caused by a single guanine deletion) (Lenis *et al.* 2010, Wang *et al.* 1994), respectively. Given that the mutation sites are known, identifying the SSR markers located near these regions that are suitable for MAS will be essential to facilitating efficient and accurate breeding.

## Soybean cyst nematode (SCN) resistance

The soybean cyst nematode (*Heterodera glycines* Ichinohe) is one of the most damaging soybean pests. In Japan, races 1, 3, and 5 of SCN have been reported. Three major loci, *rhg1*, *rhg2*, and *Rhg4*, are well known to confer resistance to soybean cyst nematodes. The *rhg1a* allele derived from ‘Peking’ encodes *SNAP18*, a soluble NSF attachment protein (Liu *et al.* 2017), whereas *Rhg4* encodes *SHMT08*, a serine hydroxymethyltransferase (Liu *et al.* 2012). The interaction between these loci modulates resistance strength. The *rhg1* locus exhibits copy number variation (CNV). The *rhg1b* allele from ‘PI 88788’, with 10 copies, confers strong resistance independently (Cook *et al.* 2012). However, resistance breakdown is a concern, prompting interest in the *rhg1a* and *rhg2* combination found in ‘PI 90763’ (Basnet *et al.* 2022). The strongest candidate gene for *rhg2* is *GmSNAP11* (Lakhssassi *et al.* 2017). Most race 3 resistance alleles in Japanese cultivar are derived from ‘Gedenshirazu’, a landrace from the Akita prefecture. In contrast, ‘PI 84751’ has been used as a genetic source of resistance to races 1 and 3. The genotype of ‘Gedenshirazu’ is *rhg4*/*rhg1-g*/*rhg2-g*, whereas that of ‘PI 84751’ is *Rhg4*/*rhg1-s*/*rhg2-s* (Suzuki *et al.* 2012). *Rhg4*, *rhg1-s*, and *rhg2-g* or *rhg2-s* are necessary for race 1 resistance, and either *rhg1-g* and *rhg2-g* or *rhg2-s*, or *rhg1-s* and *rhg2-g* or *rhg2-s* is necessary for race 3 resistance. Whole-genome sequencing of ‘Suzuhime’ (PI 494182) derived from a cross between ‘PI 84751’ and ‘Koganejiro’, revealed three candidate genes for race 1 resistance: *GmSNAP18* (*rhg1-s*), *GmSNAP11*(*rhg2-s*), and *GmSHMT08* (*Rhg4*) (St-Amour *et al.* 2020). The *GmSNAP02* is associated with race 2 resistance in ‘PI 90763’ and ‘PI 437654’, and confers a unique mode of resistance to SCN through loss-of-function mutations that implicate *GmSNAP02* as a nematode virulence target (Usovsky *et al.* 2023). Resistance to races 1 and 3 from ‘PI 84751’ was introduced into ‘Yukihomare’, a leading variety in Hokkaido, by MAS using DNA markers for *rhg1-s* and *Rhg4* and ‘Yukihomare R’ was developed (Suzuki *et al.* 2017). ‘Suzumaru R’, a suitable variety for natto, was developed by recurrent back-crossing with the donor parent ‘Chukou 1901’, using MAS with DNA markers linked to *Rhg4*, *rhg1-s*, and *rhg2-g* to introduce resistance to races 1 and 3 into the genetic background of ‘Suzumaru’ (Kurosaki *et al.* 2017). The production areas of ‘Yukihomare R’ and ‘Suzumaru R’ were 8,459 and 1,383 ha in 2023, respectively. They account for 21.6% of soybean production area in Hokkaido.

## *Phytophthora* stem and root rot (PSR) resistance

Soil-borne Oomycete *Phytophthora sojae* causes one of the most serious diseases in Japan. Although various control measures for this disease have been proposed (Dorrance 2018, Sugimoto 2013), the most cost-effective and easily adoptable strategy is the cultivation of resistant cultivars. Generally, resistance to PSR was classified into complete (qualitative) resistance through *Rps* genes and field (quantitative) resistance (Jia and Kurle 2007, Otolakoski and Huzar-Novakowiski 2024). When the pathogen infects host plants, *Phytophthora sojae* emits effector proteins encoded by avirulence (Avr) genes. In soybean varieties with complete resistance, the corresponding *Rps* gene recognizes these effectors and triggers a defensive response. In contrast, if the soybean variety is susceptible, or if there is a mismatch between the pathogen’s *Avr* gene and the host’s *Rps* gene, disease develops (Hou *et al.* 2023). More than 40 *Rps* genes have been reported (Lin *et al.* 2022). *Rps1d* and *Rps1k* are the most effective resistance genes among the 14 *Rps* genes examined using 109 *P. sojae* isolates from 14 regions in Japan (Moriwaki 2010). Although resistance is believed to be conferred by nucleotide-binding site leucine-rich repeat (NBS-LRR)-type proteins, many causal genes remain unidentified due to the highly repetitive structure of these loci. However, pan-genome analysis and complementation tests confirmed that *Rps11* is an NBS-LRR gene (Wang *et al.* 2021b). Stacking multiple *Rps* genes is considered the most effective strategy for durable resistance (Sahoo *et al.* 2021). In Japan, soybean varieties such as ‘Waseshiroge’ (*RpsWA*) (Sugimoto *et al.* 2011) and ‘Tosan 231’ (Matsuoka *et al.* 2021) have been reported to possess potentially novel race-specific resistance. These cultivars exhibit resistance to domestic pathotypes of PSR and thus hold promise for future use in resistance gene stacking strategies.

Although race-specific resistance by certain *Rps* genes has been effective for many years, field resistance is also an important complement to *Rps*-mediated complete resistance. It is broader than resistance dependent on a single *Rps* gene, and field resistance does not negatively affect yield under conditions where PSR infection does not occur (Dorrance 2018). In addition, because partial resistance does not exert strong selective pressure on the pathogen population, it is considered more durable. de Ronne *et al.* (2022) reported a novel major QTL that markedly enhanced the resistance to a broad range of pathotypes of *P. sojae* on chromosome 15. ‘Conrad’ is a representative cultivar with field resistance, and more than 35 QTLs or genomic regions have been reported in different biparental populations (Chandra *et al.* 2022). Sugimoto (2013) reported a major domestic variety, ‘Fukuyutaka’, possesses field resistance. Although the genetic basis of resistance in the cultivars is still under investigation and no resistant varieties have yet been developed using DNA markers, further progress is highly anticipated.

## Bacterial pustule resistance

Soybean bacterial pustule, caused by *Xanthomonas axonopodis* pv. *glycines*, is a serious foliar soybean disease that occurs predominantly in warm and humid climates (Sinclair 2015). The disease results in both yield and quality losses; infection can reduce seed number and size, leading to yield losses of 60% in severe cases (Kim *et al.* 2022, Sinclair 2015). Effective control of this disease involves the use of resistant varieties, and the resistance gene, *rxp*, has been successfully used in breeding programs to confer strong resistance (Hartwig and Lehman 1951). The *Rxp* gene encodes a transcription factor and the resistance allele, *rxp*, harbors a mutation that results in a truncated protein (Taguchi-Shiobara *et al.* 2024). This resistance allele is widely used in North American varieties, but rarely in Japanese varieties. Given the increasing incidence of disease in central and northern Japan owing to rising temperatures and frequent extreme weather events, the introduction of resistance genes in Japanese varieties is essential for the future stability of soybean production.

‘Suzuotome 2 gou’ is a newly developed soybean variety in which resistance to bacterial pustule has been successfully introduced. The recurrent parent variety, ‘Suzuotome’, had been cultivated primarily in Kyushu area, and is characterized by small seed size and suitability for natto processing. One of the major limitations of ‘Suzuotome’ is its high susceptibility to bacterial pustule, which frequently causes premature defoliation and yield reduction in the Kyushu region. To overcome this challenge, ‘Suzuotome 2 gou’ was developed through backcrosses four times using MAS with SSR markers flanking the *rxp* gene using a resistant variety, ‘Suzukaren’, as a donor parent. ‘Suzuotome 2 gou’ retains the agronomic and quality characteristics of ‘Suzuotome’, including early maturity, small seed size, and natto processing suitability, while the resistance and yield in ‘Suzuotome 2 gou’ are superior.

## Soybean mosaic virus (SMV) resistance

SMV causes mosaic discoloration and deformation of leaves and mottling of the seed coat, resulting in reduced seed yield and quality, respectively. This virus is transmitted both by aphids and through infected seeds, making chemical control with pesticides alone insufficient for its eradication (Hill 2015). Six strains (A, A_2_, B, C, D, and E) have been reported based on their pathogenicity in Japanese test varieties. Geographical differences were observed in the occurrence of each strain. For example, strains A and B are distributed throughout Japan, whereas strains C and D are mainly found in Kanto, Hokuriku, and the middle and southern parts of the Tohoku region (Hashimoto and Nagasawa 1987, Takahashi *et al.* 1980). Although the strain A_2_ has been reported in some regions of western Japan, the strain E, which causes severe symptoms including necrosis, has rarely been observed (Saruta *et al.* 2005). Most Japanese soybean varieties are resistant to strains A and B; however, many varieties are susceptible to strains C and D. Therefore, resistance to strains C and D should be incorporated into breeding programs that target regions where these strains are prevalent.

Canadian soybean variety ‘Harosoy’ has been identified as a resistant donor to SMV strains C and D (Takahashi *et al.* 1980). ‘Dewamusume’ is the first variety developed using this resistance allele (Ishikawa *et al.* 1979). Since then, successive resistant lines have been developed, primarily through mass selection based on large-scale inoculation using a spray gun. However, Kato *et al.* (2016) demonstrated that resistance to strains C and D, derived from the cultivar ‘Harosoy’, is conferred by *Rsv3*, which encodes a CC-NBS-LRR protein (Tran *et al.* 2018). Utilizing flanking SSR markers as selection tools, Kato *et al.* (2016, 2017) conducted recurrent backcrossing with MAS and successfully developed promising lines, such as ‘Tohoku 169’ and ‘Tohoku 173’.

Two other major loci, *Rsv1* and *Rsv4*, are also associated with SMV resistance. *Rsv1* has 10 known alleles, one of which (*Rsv1-y*) is recognized as a separate locus, *Rsv5* (Klepadlo *et al.* 2017). The *Rsv1* region forms a gene cluster and its causal gene has not been definitively identified, although CC-NBS-LRR-type proteins have been implicated (Wu *et al.* 2019). *Rsv4* functions via a distinct mechanism involving a nuclease that infiltrates the SMV replication complex and degrades double-stranded RNA, thereby suppressing viral replication (Ishibashi *et al.* 2019). Although ‘Peking’, a genetic resource serving as the *Rsv4* donor, had not been often utilized previously owing to its inferior agronomic traits, the development of DNA markers has facilitated its incorporation through MAS. In general, NBS-LRR proteins suppress disease development through a strain-specific hypersensitive response. However, because viral mutations may overcome this resistance (Kobayashi *et al.* 2014), utilizing multiple resistance genes in domestic breeding programs is favorable. ‘Peking’ has black, small, and flattened seeds, and thus the *Rsv4* gene has rarely been utilized in Japanese breeding programs where seed appearance quality is critical. However, identification of the *Rsv4* gene has enabled the development of effective DNA markers, leading to increased breeding use in recent years. ‘Haregokoro’ was developed by introducing resistance to SMV conferred by ‘Peking’ as well as those to peanut stunt virus (PSV) and southern bean mosaic virus (SBMV), into the leading cultivar ‘Sachiyutaka A1 gou’ by recurrent back crossing with MAS. It was adopted as a recommended cultivar in Okayama prefecture in 2023.

## Other virus resistance

Peanut stunt virus (PSV) causes yield reduction and seed quality deterioration owing to mottling in southwest Japan. Genetic analysis of resistance to two PSV isolates (PSV-K and PSV-T) revealed that resistance in cultivars such as ‘Hyuga’, ‘Harosoy’, and ‘Tsurunotamago 1’ is governed by a single dominant gene located at the same locus (Saruta *et al.* 2012). This resistance gene, designated *Rpsv1*, was mapped near the SSR marker Satt435 on chromosome 7 using RILs of ‘Hyuga’ × ‘Enrei’.

The soybean dwarf virus (SbDV) affects soybean production, particularly in Hokkaido and Aomori prefectures. Genetic studies have identified the Indonesian cultivar ‘Wilis’ as a source of resistance, leading to the discovery of a major resistance gene, *Rsdv1* (Uchibori *et al.* 2009). This gene was fine-mapped to a 44-kb region between the SSR markers Sat_11 and Sct_13 on chromosome 5 (Yamashita *et al.* 2013). Near-isogenic lines (Rsdv1-NILs) incorporating *Rsdv1* showed strong resistance to SbDV under both greenhouse and field conditions. In addition, Kato *et al.* (2017) developed seven elite soybean lines, including ‘Tohoku 174’, by introducing the *Rsdv1* into ‘Ohsuzu’, a major cultivar in Aomori, through recurrent backcrossing. These lines exhibited resistance under field conditions in Hokkaido, which suggests that *Rsdv1* was effective not only in the genetic background of varieties in Hokkaido but also in those adapted to the Honshu region.

The *Raso1* from cultivar ‘Adams’ confers antibiosis resistance to the foxglove aphid (*Aulacorthum solani*), a major vector of SbDV while ‘Adams’ suffers SbDV infection to a limited extent. Fine-mapping localized *Raso1* to a 63-kb region on chromosome 3, which included a nucleotide-binding site-leucine-rich repeat (NBS-LRR) gene and two additional candidate genes (Ohnishi *et al.* 2012). Introduction of *Raso1* into the susceptible cultivar ‘Toyomusume’ resulted in strong aphid resistance across all backcross lines, but was insufficient to confer SbDV tolerance.

## Common cutworm (CCW) resistance

The common cutworm (*Spodoptera litura* Fabricius) is a major lepidopteran soybean pest that causes serious damage to leaves and pods, significantly affecting plant growth and yield (Komatsu *et al.* 2010, Yadav *et al.* 2023). Resistance to lepidopteran insects has been classified into two categories: antixenosis and antibiosis, and different resistance genes are involved in each mechanism (Rector *et al.* 1999, 2000). Several resistance genetic resources against herbivorous, ‘IAC100’, ‘Soden-daizu’ (PI 229358), ‘Miyako white’ (PI 227687) and ‘Kosamame’ (PI 171451) have been identified and used in breeding programs in the United States (Cooper and Hammond 1999, Hatchett *et al.* 1976, Lambert and Kilen 1984). In Japan, a natto cultivar ‘Suzukaren’ with moderate resistance to CCW was developed from the progeny derived from the cross of ‘Suzuotome’ and resistant Brazilian variety ‘IAC 100’ (Takahashi *et al.* 2013).

In the selection of resistant lines, field-based screening may vary in accuracy depending on the year and environmental conditions. Therefore, the development and application of DNA markers are being advanced to enable more stable and efficient selection. Two QTLs located on chromosome 7, *CCW-1* and *CCW-2*, were identified through antibiosis evaluation using a population derived from a cross between ‘Fukuyutaka’ and a resistant variety in Japan, ‘Himeshirazu’, and have been incorporated into soybean breeding efforts in Japan (Komatsu *et al.* 2005, 2010). Their effects were validated by comparing ‘Fukuyutaka’ and their near-isogenic lines ‘NIL-*CCW1* + *CCW2*’ into which two resistance alleles derived from ‘Himeshirazu’ had been introduced, and the insect growth indexes (defined as pupal weight divided by the duration of the sixth instar) of ‘NIL-*CCW1* + *CCW2*’ were found to be 50% lower than those of ‘Fukuyutaka’ (Komatsu *et al.* 2005). ‘Fukuminori’ was the variety with resistance to CCW selected from ‘NIL-*CCW1* + *CCW2*’ lines, and its important agricultural characteristics, *e.g.*, maturity, yield, protein content and the processability for tofu, were almost the same as those of ‘Fukuyutaka’ (Takahashi *et al.* 2017). However, the resistance level of ‘Fukuminori’ is clearly inferior to that of ‘Himeshirazu’ (Takahashi *et al.* 2017), and thus more effort for pyramiding the CCW resistance genes will be needed for significant reductions in pesticide application or yield improvement.

Research is increasingly focused on identifying the genetic factors associated with antixenosis resistance. Notably, the QTLs *qRslx1* and *qRslx2* on chromosomes 7 and 12, respectively, were derived from a cross between ‘Fukuyutaka’ and ‘Himeshirazu’ (Oki *et al.* 2012). However, because the position of *qRslx1* was similar to that of *CCW-1* and the resistant alleles of *qRslx1* and *qRslx2* were derived from Himeshirazu and Fukuyutaka, respectively, these QTLs have not been effectively utilized for pyramiding in combination with *CCW-1* and *CCW-2*. An antixenosis resistance QTL from wild soybean (*G. soja*) collected in Kumamoto prefecture was identified at almost the same position as *CCW-2* on chromosome 7 (Oki *et al.* 2019). In contrast, two antixenosis resistance QTLs, *qRslx3* and *qRslx4*, have been discovered on chromosomes 7 and 2, respectively, in wild soybeans collected in Hiroshima prefecture (Oki *et al.* 2017). The resistant alleles of these QTLs are derived from wild soybeans and are located on chromosomes that differ from those of *CCW-1* and *CCW-2*. The incorporation of these QTLs into breeding programs is expected to contribute to the development of cultivars with enhanced resistance to common cutworms.

## Flooding tolerance

Waterlogging stress in soybeans can occur at any growth stage; however, early stage waterlogging, particularly during germination, has a disproportionately negative effect on yield. This is particularly critical in Japan, where soybean production in converted paddy fields often exceeds that in upland areas. Sayama *et al.* (2009) identified QTLs on chromosomes 8 and 12 that significantly affected germination and seedling rates, respectively. Soil hypoxia, a major consequence of waterlogging due to limited oxygen diffusion, remains a primary challenge. QTL analysis of root development under hypoxic conditions using RILs from ‘Tachinagaha’ and the tolerant landrace ‘Iyodaizu’ confirmed stable QTLs for root length (*Qrld-12*) and surface area (*Qrsad-12*) on chromosome 12 over multiple years (Nguyen *et al.* 2017).

A selection method for flooding tolerance at the flowering stage was developed, where plants were subjected to flooding in the field. ‘Shokukei-32’, a breeding line from the Hokkaido Research Organization with superior waterlogging tolerance, was developed using this method (Kousaka *et al.* 2013). The *qFTA2–1*, a QTL associated with flooding tolerance at the flowering stage, was detected on chromosome 2 using a population derived from a cross between ‘Shokukei-32’ and the flooding-sensitive variety ‘Toyoharuka’, and is effective specifically in the cross with ‘Toyoharuka’ (Kurosaki *et al.* 2025).

## Chilling tolerance

Chilling tolerance of soybeans is a critical trait for stable production in Hokkaido, the northernmost island of Japan. After the onset of flowering, yellow soybean seeds are damaged by low temperatures, leading to seed coat deterioration, including pigmentation and cracking around the hilum (Morrison *et al.* 1998, Srinivasan and Arihara 1994, Takahashi 1997, Takahashi and Asanuma 1996). This phenomenon is known as cold-induced seed coat discoloration (CD). CD is most likely caused by inhibition of CHS silencing by low temperatures (Kasai *et al.* 2009). A CD-tolerant yellow hilum cultivar, ‘Toyoharuka’, possesses a polymorphic *GmIRCHS* structure, which has been designated as the *Ic* (Kasai *et al.* 2009, Yamaguchi *et al.* 2015). *Ic*, a novel allele of the *I* locus, inhibits pigmentation of the hilum and entire seed coat. The *Ic* allele is a major gene for CD tolerance, and a DNA marker distinguishing between *I* and *Ic* alleles, named the “*Ic* marker”, has been shown to be effective for the selection of CD-tolerant soybean plants (Ohnishi *et al.* 2011). The *Ic* allele was highly effective in a field with severe cold weather damage (Yamaguchi *et al.* 2019a).

Chilling temperatures result in the appearance of cracked seeds in soybean crops, particularly in those grown in eastern and northern Hokkaido. The coats of the cracked seeds are severely split on the dorsal side, and the cotyledons are exposed and frequently separated. A stable QTL associated with seed cracking (SC) tolerance was identified in the proximal region of the *I* locus, and the *IcIc* allele promoted a stronger SC tolerance (Yamaguchi *et al.* 2015). Proanthocyanidin accumulation on the dorsal side of the seed coat, controlled by the *I* locus, leads to cracked seeds; and that homozygous *IcIc* alleles confer SC tolerance (Senda *et al.* 2018).

Histological and texture analyses of the seed coat revealed that the ability to maintain hardness and flexibility under low temperature contributes to SC tolerance in ‘Toyomizuki’, an SC-tolerant cultivar with the *II* allele (Yamaguchi *et al.* 2023). A QTL associated with SC tolerance in ‘Toyomizuki’, was detected on the chromosome 8, but the distance between this QTL and *I* locus was estimated to be 2–3 Mb. The marker analysis revealed that ‘Toyomadoka’ has the ‘Toyomizuki’ allele at this QTL, indicating that MAS for the ‘Toyomizuki’ allele will be effective (Kobayashi *et al.* 2020).

Tawny-pubescent soybeans, controlled by the dominant *T* allele at the *T* locus encoding flavonoid 3ʹ-hydroxylase, show superior chilling tolerance compared with gray-pubescent soybeans with the recessive *t* allele (Funatsuki and Ohnishi 2009). Chilling tolerance conferred by *T* is caused by the increasing antioxidant activity of 3ʹ,4ʹ-dihydroxylated flavonol derivatives (Toda *et al.* 2011). In RILs derived from the chilling-tolerant cultivar ‘Hayahikari’ and chilling-sensitive cultivar ‘Toyomusume’, a QTL detected on chromosomes 6 was directly associated with the *T* locus (Funatsuki *et al.* 2005).

The pubescence color gene (*T*) is associated with the suppression of low-temperature-induced seed coat deterioration (Takahashi 1997, Takahashi and Asanuma 1996). *T* is also effective in the suppression of SC in the *Ic* background, indicating that gene pyramiding of *Ic* and *T* contributes to high SC tolerance (Yamaguchi *et al.* 2021c). The combination of *I* and *T* alleles darkens the yellow seed coat, leading to a dirty seed appearance; however, seeds harvested from near-isogenic lines with the *IcIc*/*TT* and *IcIc*/*tt* alleles looked similar to each other and did not have a dull appearance. Therefore, the problem arising from the introduction of *T* into yellow hilum cultivars may be overcome by using the *Ic* allele.

## Genomic selection (GS) in soybean breeding program

The advent of next-generation sequencing (NGS) technologies has drastically transformed SNP analysis by enabling rapid and comprehensive decoding of whole-genome sequences. For traits such as yield, which are controlled by complex interactions among numerous genes, GS utilizing genome-wide SNP data has been applied across various crop species. For soybeans, efforts to develop GS-based breeding strategies to enhance the efficiency of selection for yield and other agronomically important traits in other countries are underway. Although no published studies that clearly document the commercial use of GS in soybean breeding by private companies in the United States or other countries have been identified, several cases have been reported at university and public research institutions in the United States; for example, Smallwood *et al.* (2019) applied GS models to predict yield, protein content, oil content, and fatty acid composition. Although yield prediction proved to be challenging, the models achieved high prediction accuracy for oleic and linolenic acid contents. Bandillo *et al.* (2023) compared genomic and phenotypic selection for yield improvement. Their results demonstrated that genomic selection outperformed phenotypic selection at high selection stringency (10–20%). Miller *et al.* (2023) developed GS models for yield, protein, and oil content using a training population from the soybean breeding program at the University of Georgia, which targets commercial cultivar development. Similar to the findings of Smallwood *et al.* (2019), the yield was difficult to predict; however, moderate prediction accuracy was achieved for protein and oil content.

The SoyaGen project is a collaborative research initiative launched in Canada to accelerate soybean genetic improvement through genomics-derived solutions funded by Genome Canada and other national and provincial partners (Belzile *et al.* 2022). The SoyaGen project brings together academic researchers, breeders, and industry stakeholders. The main objectives include (1) the development of genomic prediction models for agronomic traits such as yield, maturity, disease resistance, and seed composition; (2) the identification and deployment of disease resistance loci, particularly for *Phytophthora* root rot and soybean cyst nematodes; and (3) the integration of GS into public and private breeding programs. Notably, the project demonstrated that GS can outperform traditional phenotypic selection under certain conditions, and that GS-based parental selection in actual breeding pipelines led to higher frequencies of elite progeny using high-throughput genotyping platforms and extensive historical breeding data (Jean *et al.* 2021). SoyaGen also developed decision-support tools and MAS strategies that have already been adopted by breeding programs in Canada. This project represents one of the first real-world implementations of GS in soybeans, offering a model for genomics-enabled breeding that can be adapted to other crops and regions.

The Strategic Innovation Promotion Program Phase 3 (SIP3) has launched a national project aimed at establishing a robust breeding platform and advanced cultivation technologies to support the stable and efficient production of high-quality soybean protein in Japan (Secretariat of Science, Technology and Innovation Policy, Cabinet Office 2023). In response to the growing global demand for plant-based proteins, driven by food security concerns and environmental sustainability, Japan has positioned soybean, a key source of plant protein, as a strategic crop. Conventional Japanese soybean breeding programs face several issues such as the limited development of high-yield cultivars with adaptation to food processing and consumer needs, vulnerability to recent extreme climate change, and deterioration in cultivation management due to labor shortages. To overcome these issues, SIP3 seeks to establish an integrated breeding and cultivation framework that leverages cutting-edge genomic and digital technologies, fostering innovation from breeding to on-farm implementation through industry-academia collaboration. As enhancing soybean yield in Japan has become an urgent priority to increase self-sufficiency and strengthen national food security, an integrated analysis-based breeding platform based on genomic information will be established to enable public research institutions and companies to breed more efficiently.

## Future prospects

As exemplified by the Canadian breeding model, constructing a new breeding platform capable of selecting optimal crossing combinations is anticipated to contribute substantially to breeding efficiency. For example, when attempting to develop high-yielding cultivars by crossing varieties that differ in their composition of flowering-related genes, segregation of those genes in the hybrid progeny may cause undesirable variations in traits such as maturity, plant height, and biomass. This noise interferes with the accumulation of yield-enhancing alleles. Such issues are particularly problematic when incorporating yield-related genes from overseas high-yielding cultivars that are genetically divergent from the Japanese varieties. Conversely, eliminating crossing combinations that lack agriculturally essential traits, such as pod shattering and disease resistance, or selecting combinations in which such traits do not segregate in the progeny are advantageous. Achieving this necessitates cataloging the allele compositions of flowering-related and other agriculturally essential genes in the breeding materials under consideration. Advances in NGS technology are expected to play a key role in supporting this process. In future, if the allelic compositions of a wide range of breeding materials are cataloged and predictive tools for segregation in hybrid progeny are developed, it will be possible to eliminate crossing combinations that result in the segregation of flowering-related genes or the absence of essential agronomic traits. This will significantly enhance the overall efficiency of breeding programs in Japan.

## Author Contribution Statement

A.K. drafted the entire manuscript. N.Y. and S.K. wrote the sections of manuscript related to the breeding in the Hokkaido and Honshu region, respectively. All authors read and approved the final version.

## Supplementary Material

Supplemental Table
